# Medicines Acceptability in Hospitalized Children: An Ongoing Need for Age-Appropriate Formulations

**DOI:** 10.3390/pharmaceutics12080766

**Published:** 2020-08-13

**Authors:** Thibault Vallet, Omar Elhamdaoui, Amina Berraho, Lalla Ouafae Cherkaoui, Yamna Kriouile, Chafiq Mahraoui, Nezha Mouane, Anne-Marie Pense-Lheritier, Fabrice Ruiz, Yahya Bensouda

**Affiliations:** 1 ClinSearch, 110 Avenue Pierre Brossolette, 92240 Malakoff, France; fabrice.ruiz@clinsearch.net; 2 Faculty of Pharmacy and Medicine, Mohammed V University in Rabat, Impasse Souissi, 10170 Rabat, Morocco; omar.elhamdaoui@um5s.net.ma (O.E.); a.berraho@um5s.net.ma (A.B.); o.cherkaoui@um5s.net.ma (L.O.C.); y.kriouile@um5s.net.ma (Y.K.); c.mahraoui@um5s.net.ma (C.M.); n.mouane@um5s.net.ma (N.M.); y.bensouda@um5s.net.ma (Y.B.); 3 Specialties Hospital, University Medical Centre Ibn Sina (CHIS), Quartier Souissi, 10170 Rabat, Morocco; 4 Pediatrics Hospital, University Medical Centre Ibn Sina (CHIS), Avenue Ibn Rochd, 10100 Rabat, Morocco; 5 EBInnov, Ecole de Biologie Industrielle (EBI), 49 Avenue des Genottes, 95800 Cergy-Pontoise, France; am.lheritier@hubebi.com

**Keywords:** medicine, acceptability, swallowability, tablet, pediatric, children, formulation, hospital, clinsearch acceptability score test (CAST)

## Abstract

Although knowledge on medicine acceptability remains fragmented, this multi-faceted concept has emerged as a key factor for compliance in pediatrics. In order to investigate the acceptability of medicines used in the University Medical Centre Ibn Sina (CHIS) of Rabat, Morocco, an observational study was conducted. Using a multivariate approach integrating the many aspects of acceptability, standardized observer reports were collected for 570 medicine intakes in patients up to the age of 16, then analyzed on a reference framework. Tablets appeared to be well accepted in children greater than 6 years old, but were crushed/dissolved for 90% of the 40 children aged from 3 to 5, and 100% of the 38 patients younger than 3. Moreover, the prescribed dose was fully taken for only 52% and 16% of these younger children, respectively. Despite this, tablets represented 24% of evaluations in children from 3 to 5 and 20% in infants and toddlers. Oral liquid preparations appeared to be better accepted than tablets in preschoolers, but not for those under 3. Overall, these findings highlight the lack of suitable alternatives for the younger children, especially for formulations of antiepileptics, antithrombotic, and psycholeptic agents in the local context.

## 1. Introduction

Prescribing well accepted formulations is of the upmost importance for the pediatric population. Acceptability, defined as the overall ability and willingness of the patient to use and its care giver to administer the medicine as intended, has emerged as a key factor for treatment effectiveness as it may negatively impact patient adherence [1]. Indeed, children unwilling to take an oral medication due to a poor taste, run the risk of not following the recommended course of treatment [2,3]. Obviously, palatability of oral medication is crucial in pediatrics; however, the multi-faceted concept of acceptability is driven by many features of patients and products. For instance, while organoleptic issues are limited for solid oral dosage forms (SODF), tablets and capsules could be difficult for younger children to swallow. However, easy to swallow oral liquid pharmaceutical products might be related to volume and palatability issues which may vary across cultures [4]. Although knowledge has increased and healthcare stakeholders (e.g., regulators, industrials, prescribers) have become more aware of acceptability issue in recent years, much remains to be done for developing age-appropriate formulations covering all unmet therapeutic needs in pediatrics, and prescribing the right product to each patient. Indeed, dividing tablets designed for adults to reduce dosages, or crushing medications to be administrated to the younger children unable to safely swallow SODF, remain widespread practices in pediatric care which may result in critical issues regarding posology or bioavailability [5,6,7]. Referencing child friendly alternatives to frequently prescribed tablets or parenteral formulations, and promoting their good use, is crucial for ensuring treatment effectiveness and providing a high quality of care in pediatrics. Clinical pharmacists, working closely with physicians, nurses, and other healthcare professionals, play an important role in promoting patient centered-care at hospital. For this purpose, such branch of pharmacy has been strengthened in the University Medical Centre Ibn Sina (CHIS) of Rabat, Morocco. Herein, we carried out an observational study to investigate the appropriateness of the different medications administered to the local pediatric population, aiming to highlight the ongoing needs at CHIS and to improve the knowledge on factors affecting acceptability in this setting.

## 2. Materials and Methods

### 2.1. Objective, Study Design and Setting

The objective of this multicenter, prospective, cross-sectional, and strictly observational study was to investigate the acceptability of medications used in hospital for the Moroccan pediatric population. The study was carried out in the Pediatrics and the Specialties Hospitals of the University Medical Centre Ibn Sina (CHIS) of Rabat, Morocco, between November 2018 and February 2020.

The study was conducted in accordance with the ethical principles that have their origins in the Declaration of Helsinki, as well as the Moroccan legislation, which does not require any ethics approval for an observational study [8].

To perform our explorations we used the ClinSearch Acceptability Score Test^®^ (CAST) [9,10], a data-driven approach based on real-life observer-reported outcomes, as described hereafter.

### 2.2. Participants

Inclusion criteria required that patients were less than 18 years old, hospitalized, receiving any medication, with the exception of infusions in which a catheter was already present at the time of the observation. All patients answering these criteria were included without any randomization, on a voluntary basis. Verbal consent from the parent/legal guardian, and from the patient when possible was obtained.

### 2.3. Data Collection

Once enrolled in the study, a standardized web-questionnaire was completed by a trained member of the site study team observing the use of the very first medicine due to be administered at the next medication round for each participant. As such, any potential bias caused by prior or co administration of other medicines was avoided.

The researcher reported the following observable events or behaviors:

- results of intake (the required dose was fully, partly or not taken at all);
- patient reaction during the administration (positive, neutral or negative reaction);
- times needed to prepare (from opening any packaging to having a required dose of medication ready to use, including all handling and modifications), and to administer the required dose of medication (from a required dose of medication ready to use to the end of the intake). The sum of the times of preparation and administration time, both recorded using 10 s intervals, was classified as short (1 min and less), medium (from 1 min to 2 min and 30 s), or long (more than 2 min and 30 s).

Any of the following methods used to ease/achieve administration were also reported:

- dividing the intake of a dose which could not be taken as a whole;
- altering the intended use (any modifications of dosage forms such as capsules opened or tablets crushed; use of another route/mode of administration);
- using food/drink to mask a bad taste or ease swallowing;
- using an administration device not provided with the medication;
- promising a reward;
- using restraint (administration was imposed upon the patient).

For each method ticked the observer was requested to specify any further information in a text field.

Each evaluation of one medicinal product, taken by one patient, corresponded to a particular combination of these observed measures describing the many aspects of acceptability.

In addition, the researcher filled in the exact name of the medicine taken by the patient, some information on the context of use, and some characteristics of both the patient and the treatment recorded from the patient’s medical record.

### 2.4. Data Analysis

To score acceptability we used the acceptability reference framework. This tool is based on multivariate analysis mining a large set of 2366 evaluations, comprised of those from Morocco and those collected using the standardized web-questionnaire in five other countries with various cultures (FR, NO, UK, IN, and JP) in both domestic and hospital settings since May 2015 [9,10]. Diversified sources of data allow us to better grasp the multi-faceted concept of acceptability.

A mapping process, Multiple Correspondence Analysis, highlighted the key relationships between all the evaluations into a low-dimensional space: the 3D acceptability map. This analysis positioned the many evaluations as points onto the map according to their similarity, from the combination of the ideal observed measures to the worst combinations. Subsequently, a clustering process, Hierarchical Clustering on Principal Components k-means consolidation, gathered the most similar evaluations into two meaningful clusters, defining coherent acceptability profiles. The acceptability map juxtaposed green and red zones that materialize the positively and negatively accepted profiles, respectively.

The evaluations of tablet intakes from the Moroccan hospitals were partitioned in four age groups: under 3 years (infants and toddlers), from 3 to 5 (preschoolers), from 6 to 11 (grade-schoolers), 12 and over (adolescents). Each age group was positioned on the map, at the barycenter of its evaluations. If the barycenter, along with the entire 90% confidence ellipsis surrounding it, belonged to the green area of the map, the tablet could be classified as accepted in the sub-population of patients. Similarly, the acceptability of oral liquid preparations was studied in each age group (see the video abstract for an illustration of the mapping, clustering, and scoring processes).

In addition, statistical tests were used to assess the significance of the differences observed between the different acceptability scores (age groups) in term of patients’ characteristics, products’ features and observed measures composing the acceptability scores. When there was a minimum expectation of 5 for 80% of cells without any null expectation Pearson’s Chi-squared test was used, Fisher’s exact test was used instead.

## 3. Results

### 3.1. Patients and Medicines

In this study, 570 evaluations of medicine use were collected in the Moroccan hospitals. Table 1 presents the characteristics of the patients and products, stratified by patient age.

Among these 570 evaluations, 161 (28%) were of tablets, which represented 20% (*n* = 38) of evaluations in children under 3, 24% (*n* = 40) in children from 3 to 5, 36% (*n* = 59) in children from 6 to 11, and 53% (*n* = 24) in children aged 12 and over. Twenty-eight distinct medicinal products formulated as a tablet were assessed. These products were composed of 21 distinct chemical substances from 15 therapeutic areas. Most were antiepileptics (22%) and antithrombotic agents (21%).

The remaining evaluations included medications which are generally considered more appropriate for young children: ready-to-use oral liquids (33%) as well as the medications which must be dissolved/dispersed in a liquid prior to administration (39%). Only one capsule intake was assessed.

Table S1 presents the different drug formulations in each therapeutic subgroup.

### 3.2. Acceptability of Tablets

**Figure 1:**
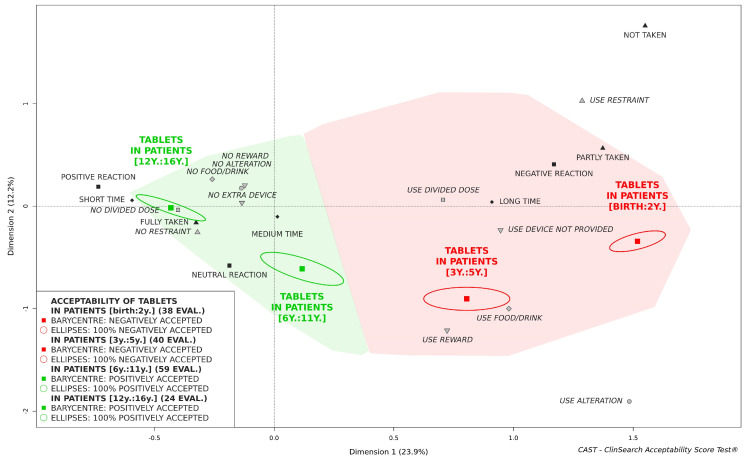
Acceptability of tablets in pediatrics depending on age.

Tablets appeared to be "positively accepted" in children aged 6 and over, while not in children under 6 (Figure 1). Indeed, the barycenters of the evaluations of tablet intake in children from 6 to 11 as well as 12 and over, along with the confidence ellipses surrounding them, were fully located in the green area of the acceptability map. This appeared not to be the case for the children from 3 to 5, or those under 3, which were fully located in the red part of the acceptability map.

The first dimension of the map juxtaposes the positively connoted categories on the left side (dimension 1 negative coordinates) to the categories that are negatively connoted on the right side (dimension 1 positive coordinates). The group of adolescents appeared to be located closest to the ideal position on the map, while the infants and toddlers group was located closest to the worst position. However, considering the insufficient number of adolescents (n < 30), we could only describe acceptability tendency in this group.

Differences in the acceptability scores reflect the significant differences observed for all of the constituting variables (Table 2). All the negatively connoted observed measures were reported more frequently in the younger children with the exception of promising a reward. This method was most used in pre-and grade-schoolers, as their willingness to take the medicine could be more easily influenced. The required dose of medication was fully taken for 100% of the children aged 12 and over, 90% of the children from 6 to 11, 52% of the children from 3 to 5, and for only 16% of the younger. The required doses not fully taken were due to tablet alterations. Indeed, the prescribed dose of the medication was modified before administration (prescribed dose of tablet crushed into powder, dissolved into a liquid or divided in several smaller pieces to be swallowed successively) for 100% of the children less than 3, 90% of the children from 3 to 5, 53% of the children from 6 to 11, and only once for the adolescent patients. Such modifications mainly resulted in using food or drink to mask a bad taste or ease swallowing, dividing the intake of a dose which cannot be taken as a whole, and using a device such as oral syringe to achieve administration.

Among the 28 distinct tablet pharmaceutical products assessed in this study, 18 (64%) were modified prior administration for at least one evaluation: 100% of the 12 products assessed in infants/toddlers, 100% of the 14 in preschoolers, 56% of the 16 in grade- schoolers, and 8% of the 13 in adolescents. Table 3 presents the different types of modifications, stratified by patient age.

### 3.3. Acceptability of Oral Liquids Pharmaceutical Products

**Figure 2:**
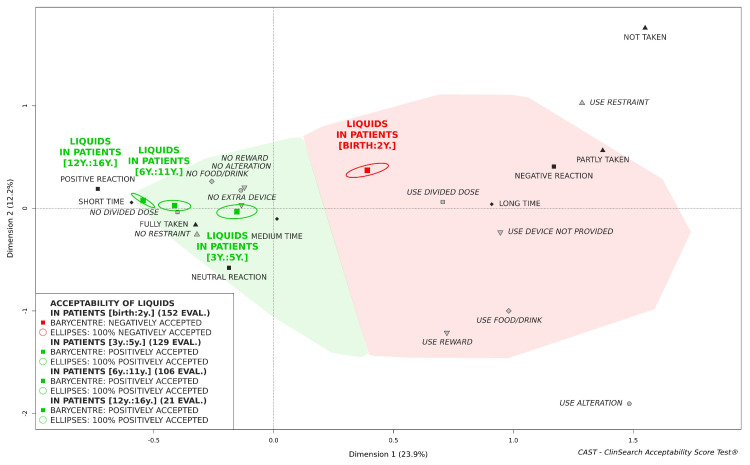
Acceptability of oral liquid pharmaceutical products in pediatrics, stratified by patient age.

Oral liquid pharmaceutical products considered as a whole, either ready-to-use or to be reconstituted, could be classified as "positively accepted" in children aged 3 and over, while not in children under 3 (Figure 2). Liquids appeared to be well accepted in children aged from 3 to 5. Although oral liquids were located farther from the worst position on the map than tablets, they remained fully located in the red area of the map for the children under 3.

Focusing on those children under 3, syrup tended to be better accepted than other oral liquid pharmaceutical products. Indeed, the barycenter of the 37 evaluations of syrup intake, along with 79% of the confidence ellipses surrounding it, were positioned in the green area of the map. Conversely, the barycenters of the 26 evaluations of effervescent tablets, 33 evaluations of powders for oral solution/suspension, 20 evaluations of drops for oral solution, and 36 evaluations of oral solutions/suspensions were all located in the red area of the map.

## 4. Discussion

Exploring the acceptability of medications used in the pediatric populations of two Moroccan hospitals, this study highlighted some issues regarding age appropriateness of oral formulations in children under 6, especially for infants and toddlers.

While tablets could be considered as accepted in children 6 years and older, that appeared not to be the case among younger children as expected. Tablets are widely manufactured, and are prescribed due to their many advantages such dosage accuracy, stability, and the ease of storage. However, the main disadvantage in pediatrics is the difficulty, or even inability, experienced by younger children attempting to swallow these dosage forms [4]. Although there are significant inter-patient differences, by 6 years of age, most children are able to safely swallow SODF [11]. Training of patients under 6 may positively impact their ability to swallow [12,13,14,15]. However, the risk of aspiration, gagging, and choking remain until the complete maturation of deglutition functions are established, generally by 6 years of age [16]. Herein, observers reported an intact tablet swallowed for only 5% of the evaluations in patients under 6. Swallowing difficulties led to manipulation of drugs such as crushing, dispersing or splitting tablets. Indeed, observers reported manipulation prior administration for 95% of tablet intakes assessed in the younger children. This problem, which may significantly affect critical factors such as dosage accuracy or drug bioavailability, continued to be observed among patients older than 6; tablets were altered for 53% of grade-schoolers. According to therapeutic subgroups, as defined by the Anatomical Therapeutic Chemical (ATC) classification system, the tablets manipulated prior administration were drugs from the antiepileptics group for 29% of evaluations: the antithrombotic agents group for 20%, and the psycholeptics group for 13%. 

Although tablets present limited organoleptic issues (e.g., a tablet coating provides a physical barrier between the drug and the patient’s taste buds), crushing, dispersing or splitting impose a direct exposure to the pharmaceutical ingredient which may generate palatability issues. Indeed, many drugs have a bitter and often aversive taste [17]. Thus, crushed tablets were mainly given with food or drinks to improve palatability and facilitate deglutition. Beyond dosage form manipulation, mixing with food or drink may have an effect on the biopharmaceutical characteristics and, consequently, on the product’s overall performance [18,19,20]. As various food and drink may have different effects on the properties of the preparation (e.g., acceptability, compatibility, and stability), harmonization of recommended co-administration strategies would be of great interest, even if cultural differences may be a limitation [1,21]. Due to children’s differences in food consumption and body composition, food-drug interactions cannot be predicted based on adult studies [20,22]. Furthermore, the dispersion of a crushed tablet in a liquid can also lead to a loss of active substance that may be adsorbed by the serving containers surface or fall out of suspension and remain behind after administration [23]. The time consuming need to manipulate a medication not available in a licensed appropriate formulation is likely to have a significantly negative impact on nursing staff resources in an hospital setting [24,25]. The risks and burden related to those practices are avoidable using child-friendly alternative to tablets. Recourse to oral liquid pharmaceutical products has been the traditional choice for pediatric patients. Indeed, facilitating dosage adaptation and ease of swallowing, such products could help prescribers, avoid SODF acceptability issues in young children. However, poor organoleptic properties are well known to negatively impact acceptability of oral liquid preparations. Thus, drug taste masking resulting in a palatable medication, remains a key factor to ensure compliance in pediatrics [2,3,26]. In this study, 88% of the oral liquid pharmaceutical products assessed were formulated with a flavor, and 93% with a flavor and/or a sweetener (data missing for one pharmaceutical product). Only 3 drugs (Haloperidol, Lactulose, and Valproic acid) among the 31 drugs formulated as oral liquid pharmaceutical products that were assessed in the study, were formulated without a flavor nor a sweetener. These findings demonstrate the designer’s cognizance of palatability issues for this kind of formulation.

Although oral liquid pharmaceutical products appeared to be better accepted than tablets in children from 3 to 5, it seemed not to be the case for those under 3. Herein, oral liquid pharmaceutical products encompass ready-to-use oral liquids (i.e., syrup, oral solutions or suspensions) as well as the reconstituted oral liquids (i.e., powders or effervescent tablets which must be dissolved or dispersed in a liquid prior to administration). The results show that syrup tended to be better accepted than other oral liquid preparations in infants and toddlers. However, such formulation may be related to other issues in the younger children. Many excipients frequently used (e.g., preservatives, sweeteners, and dyes) may be associated with toxicological risks and safety problem [27], and relevant measuring devices are crucial to prevent dosage inaccuracy, especially at home [28]. Consequently, there is still a need for further formulations suitable for younger patients. Orodispersible forms (e.g., tablet or film) have the advantage of being administered without swallowing, and may prove to be of interest. Nevertheless, flavors or sweeteners should be needed as taste and grittiness remain central considerations [4]. Klingmann et al., demonstrated that oral thin films are a suitable alternative to liquid pediatric formulations in neonates [29]. Mini-tablets could also be an alternative for future development. Indeed, several studies have demonstrated that preschoolers, toddlers, and even infants over 6 months of age could swallow at least one single mini-tablet [30,31,32]. In addition, acceptability of mini-tablets appeared to be significantly superior to syrup. However, limitations with high doses remain and an effective counting device is crucial to prevent the risk of incorrect dosing if multiple mini-tablets are required per dose [4]. Nevertheless, shifting from liquids to solid forms should be relevant regarding stability, cost, and global availability of medicines [33,34].

In view of the above and based on a clinical pharmacy approach, Table 4 suggests alternatives to those conventional SODF—tablets and capsules—for the 15 drugs formulated as tablets—18 distinct pharmaceutical products—that were modified prior administration in the study. Firstly, alternatives should be child-friendly formulations of the same active pharmaceutical ingredient (API). In the absence of such age-appropriate formulations, therapeutic or chemical equivalence could be envisaged (pharmaco-therapeutic family or chemical subgroup of the ATC classification system). Nevertheless, physicians and clinical pharmacists should always discuss the appropriateness of potential alternatives considering both the characteristics of patient (e.g., age, disabilities, and comorbidites) and products (e.g., therapeutic indication(s), strength, and excipients). No alternative formulations could be identified for five drugs (Cotrimoxazole, Efavirenz, EKIP-4K, Lamivudine, and Propranolol), while there were no alternative formulations available in Morocco for two drugs (Acenocoumarol and Sildenafil). Oral liquid preparations should be envisaged locally for only five drugs (Clobazam; Hydrocortisone; Levetiracetam; Paracetamol, Codeine; Prednisone); otherwise, formulations for parenteral use—ophthalmic for one drug—could be considered. This table shows that acceptability issues in children could be due to prescriptions which failed to adequately consider the particular needs of the targeted patient, but frequently appeared to be driven by the lack of age-appropriate formulations in the local context, especially for infants and toddlers.

Constant vigilance is required to uncover unmet needs in pediatrics and potential alternatives should be sought and discussed by working groups including all the actors of the hospital (i.e., physicians, pharmacists, nurses, administrators, and patient representatives). Such inclusive groups would best enable adequate prescriptions in pediatrics and the optimization of medicinal products referenced on hospital prescription lists, by raising awareness about the crucial role of acceptability at all levels of the health system. Obviously, such discussions should consider both therapeutic indications and pharmaco-economic issues. Indeed, the cost differences between the offending SODFs and child-friendly alternatives, the limited volume of prescriptions for young children, and some issues with continuity of supply may impose some limitations to the variety of dosage forms stocked in different local contexts, and tablet modifications may be needed as a last resort to administer the required dose of drugs to young children. Such inevitable modifications should be clearly identified by hospital staff to define a validated protocol ensuring treatment effectiveness and patient safety. Indeed, the summary of product characteristics (SmPC) explicitly permit crushing tablets for only 4 drugs among the 15 that were modified prior administration in the study. Alternatively, the hospital pharmacy could alleviate such lack of child-friendly formulations by turning some key drugs widely used in pediatrics from tablet to extemporaneous formulations suitable for use in children [35].

Comparing those results with findings from other countries should be of real interest, as many factors, such as awareness and resources driven by economic and sociocultural context, may impact the ways in which stakeholders of the health care system consider acceptability issues in vulnerable populations. However, although healthcare professionals, such as clinical pharmacists, may promote patient centered-care in a local setting, medicine acceptability in vulnerable populations remains a worldwide ongoing problem and further development of age-appropriate formulations by pharmaceutical compagnies are needed.

## 5. Conclusions

This study was an opportunity to quantify and visualize a recurring problem in pediatrics: the poor acceptability of tablets in young children. When available, recourse to oral liquid preparations is a common alternative to SODF, in an attempt to ease medicine administration. However, we objectively demonstrated that such an alternative could be sub-optimal in infants and toddlers. In this study, acceptability issues appear to be mainly due to the scarcity of child-friendly dosage forms available at the hospital. Pending the implementation of international directives aiming to address this problem on the industrial level, inclusive working groups bringing together all local stakeholders may raise awareness on potential issues related to tablet modifications, look for galenic or therapeutic alternatives already on the market, or even support the development of extemporaneous pediatric formulations for key drugs without an alternative to tablets. As a last resort, validated protocols should be established for any unavoidable dosage form modifications so as to ensure treatment safety and efficacy.

## Figures and Tables

**Table 1:** Characteristics of the patients and products included in the study, stratified by patient age.

Characteristics	Patient Age				Statistical Test
	[Birth:2y.] (*n* = 191)	[3y.:5y.] (*n* = 169)	[6y.:11y.] (*n* = 165)	[12y.:16y.] (*n* = 45)	
**Sex of patients**					χ^2 b^: *p* = 0.18
Boy	87 (46) ^a^	95 (56)	89 (54)	26 (58)	
Girl	102 (54)	74 (44)	75 (46)	19 (42)	
missing data	2		1		
**Formulations of medicines**					χ^2^: *p* < 0.001
Oral liquid preparations	152 (80)	129 (76)	106 (64)	21 (47)	
Solid oral dosage form	39 (20)	40 (24)	59 (36)	24 (53)	
**Therapeutic areas of medicines**					F ^c^: *p* < 0.001
Antibacterials	42 (22)	49 (29)	42 (25)	8 (18)	
Corticosteroids	46 (24)	41 (24)	27 (16)	5 (11)	
Antiepileptics	46 (24)	29 (17)	24 (15)	7 (16)	
Antithrombotic agents	2 (1)	10 (6)	18 (11)	4 (9)	
Antianemic preparations	17 (9)	3 (2)	3 (2)	1 (2)	
Antivirals	10 (5)	4 (2)	2 (1)	7 (16)	
Psycholeptics	5 (3)	7 (4)	8 (5)	0 (0)	
Analgesics	1 (1)	6 (4)	11 (7)	0 (0)	
Ophthalmologicals	0 (0)	1 (1)	12 (7)	1 (2)	
Muscle relaxants	1 (1)	3 (2)	4 (2)	5 (11)	
Antimycobacterials	2 (1)	4 (2)	2 (1)	2 (4)	
Mineral supplements	4 (2)	1 (1)	2 (1)	2 (4)	
Urologicals	0 (0)	0 (0)	1 (1)	2 (4)	
Others (<5%)	15 (8)	11 (7)	9 (5)	1 (2)	

^a^*n* (%): number and percentages. ^b^ χ²: Pearson’s Chi-squared Test *p*-value. ^c^ F: Fisher’s Exact Test *p*-value.

**Table 2:** Observer-reported outcomes for tablet intakes, stratified by patient age.

Outcomes	Patient Age				Statistical Test
	[Birth:2y.] (*n* = 38)	[3y.:5y.] (*n* = 40)	[6y.:11y.] (*n* = 59)	[12y.:16y.] (*n* = 24)	
**Result intake**					F ^b^: *p* < 0.001
fully taken	6 (16) ^a^	21 (52)	53 (90)	24 (100)	
partly taken	32 (84)	19 (48)	6 (10)	0 (0)	
not taken	0 (0)	0 (0)	0 (0)	0 (0)	
**Patient’s reaction**					χ^2 c^: *p* < 0.001
Positive	0 (0)	1 (2)	15 (25)	15 (62)	
Neutral	2 (5)	14 (35)	29 (49)	5 (21)	
Negative	36 (95)	25 (62)	15 (25)	4 (17)	
**Manip.-admin. time**					χ^2^: *p* < 0.001
short time	1 (3)	8 (20)	29 (50)	19 (79)	
medium time	5 (13)	21 (52)	24 (41)	5 (22)	
long time	32 (84)	11 (28)	5 (9)	0 (0)	
missing data			1		
**Divided dose**					χ^2^: *p* < 0.001
no divided dose	4 (11)	12 (30)	43 (73)	23 (96)	
use divided dose	34 (89)	28 (70)	16 (27)	1 (4)	
**Alteration**					χ^2^: *p* < 0.001
no alteration	0 (0)	4 (10)	28 (47)	23 (96)	
use alteration	38 (100)	36 (90)	31 (53)	1 (4)	
**Device not provided**					χ^2^: *p* < 0.001
no extra device	12 (32)	37 (92)	55 (93)	24 (100)	
use device not provided	26 (68)	3 (8)	4 (7)	0 (0)	
**Food drink**					χ^2^: *p* < 0.001
no food/drink	2 (5)	8 (20)	35 (59)	23 (96)	
use food/drink	36 (95)	32 (80)	24 (41)	1 (4)	
**Reward**					χ^2^: *p* < 0.001
no reward	33 (87)	8 (20)	25 (42)	20 (83)	
use reward	5 (13)	32 (80)	34 (58)	4 (17)	
**Restraint**					χ^2^: *p* < 0.001
no restraint	5 (13)	36 (90)	56 (95)	24 (100)	
use restraint	33 (87)	4 (10)	3 (5)	0 (0)	

^a^*n* (%): number and percentages. ^b^ F: Fisher’s Exact Test p-value. ^c^ χ²: Pearson’s Chi-squared Test *p*-value.

**Table 3:** Type of tablet modification, stratified by patient age.

Patient Age	Crushed into Powder	Dissolved into Liquid	Divided in Smallest Pieces	Unspecified
[Birth:2y.] (*n* = 38)	28 (74) ^a^	7 (18)	1 (3)	2 (5)
[3y.:5y.] (*n* = 36)	17 (47)	11 (31)	4 (11)	4 (11)
[6y.:11y.] (*n* = 31)	12 (39)	5 (16)	9 (29)	5 (16)
[12y.:16y.] (*n* = 1)	1 (100)	0 (0)	0 (0)	0 (0)

^a^*n* (%): number and percentages.

**Table 4:** Suggestions of alternatives to conventional solid oral dosage forms (SODF) for drugs formulated as tablets and modified prior administration in the study.

Drugs	Alternatives to Conventional SODF	
	Equivalence	Dosage Form
Acenocoumarol	INN ^a^	Mini-Tablet ^c^
	ATC ^b1^: B01AA (Warfarine)	Oral suspension ^c^
Acetazolamide	INN	Powder and solvent for injection solution ^c,d^
	ATC: S01EC (Dorzolamide; Brinzolamide; Brimonidine)	Ophthalmic solutions
Baclofen	INN	Oral solution ^c^; Solution for perfusion ^c^
	ATC: M03BX (thiocolchicoside)	Solution for injection
Clobazam	INN	Oral suspension
	ATC: N05BA (Prazepam)	Drops Oral solution
Hydrocortisone	INN	Lyophilisat and solution for parenteral use ^e^
	Pharmaco-Therapeutic ^b2^: steroidal anti-inflammatory drugs (Ketotifen)	Syrup; Oral solution
	ATC: H02AB (Dexamethasone; Prednisolone; methylprednisolone)	Syrup; Oral solution; Powder for injection solution
Levetiracetam	INN	Oral solution; Solution for perfusion; Coated Granules ^c^
	ATC: N03AX (Lamotrigine)	Dispersible tablet
Paracetamol, Codeine	INN	Effervescent tablet
	Pharmaco-Therapeutic: analgesic, antipyretic (Paracetamol)	Oral solution
Phenobarbital	INN	Powder and solvent for injection solution ^e^
Prednisone	Pharmaco-Therapeutic: steroidal anti-inflammatory drugs (Ketotifen)	Syrup; Oral solution
	ATC: H02AB (Dexamethasone; Prednisolone)	Syrup; Oral solution
Sildenafil	INN	Oral suspension ^c^

^a^ Pharmaceutics alternative: other dosage form for the same International Nonproprietary Name (INN). ^b^ Therapeutic alternative: other dosage form for: ^1^ the same chemical subgroup of the Anatomical Therapeutic Chemical (ATC) classification system; ^2^ the same pharmaco-therapeutic family. ^c^ not available in Morocco. ^d^ cost issue. ^e^ issue related to the continuity of supply.

## Supplementary Materials

The following are available online at https://www.mdpi.com/1999-4923/12/8/766/s1, Table S1: Therapeutic areas and formulations of drugs for the 570 evaluations collected in the Moroccan hospitals, stratified by patient age.
