# Supplementary material for: Medicines Acceptability in Hospitalized Children: An Ongoing Need for Age-Appropriate Formulations
Source: Pharmaceutics. 2020 Aug 13;12(8):766. doi: 10.3390/pharmaceutics12080766 (PMC7463451; doi:10.3390/pharmaceutics12080766)
Supplement: Supplementary file 1 [file pharmaceutics-12-00766-s001.pdf]

pharmaceutics

Supplementary Materials:Medicines Acceptability in Hospitalized Children: an Ongoing Need for Age-Appropriate Formulations

Thibault Vallet [1,*], Omar Elhamdaoui [2,3], Amina Berraho [2,3], Lalla Ouafae Cherkaoui [2,3], Yamna Kriouile [2,4], Chafiq Mahraoui [2,4], Nezha Mouane [2,4], Anne-Marie Pense-Lheritier [5], Fabrice Ruiz [1], Yahya Bensouda [2,3]

1 ClinSearch, 110 Avenue Pierre Brossolette, 92240 Malakoff, France; fabrice.ruiz@clinsearch.net
2 Faculty of Pharmacy and Medicine, Mohammed V University in Rabat, Impasse Souissi, 10170 Rabat, Morocco; omar.elhamdaoui@um5s.net.ma (O.E.); a.berraho@um5s.net.ma (A.B.); o.cherkaoui@um5s.net.ma (L.A.C.); y.kriouile@um5s.net.ma (Y.K.); c.mahraoui@um5s.net.ma (C.M.); n.mouane@um5s.net.ma (N.M.); y.bensouda@um5s.net.ma (Y.B.)
3 Specialties Hospital, University Medical Centre Ibn Sina (CHIS), Quartier Souissi, 10170 Rabat, Morocco
4 Pediatrics Hospital, University Medical Centre Ibn Sina (CHIS), Avenue Ibn Rochd, 10100 Rabat, Morocco
5 EBInnov, Ecole de Biologie Industrielle (EBI), 49 Avenue des Genottes, 95800 Cergy-Pontoise, France; am.lheritier@hubebi.com
* Correspondence: thibault.vallet@clinsearch.net; Tel.: +33(0) 1 47 35 17 17 (T.V.)

Received: 30 June 2020; Accepted: 10 August 2020; Published: date

Table 1. Therapeutic areas and formulations of drugs for the 570 evaluations collected in the Moroccan hospitals, stratified by patient age.

Therapeutic Areas	Drugs	Formulations	Evaluations (N)	Distrubution By Age Group (%)			
				[Birth:2y.]	[3y.:5y.]	[6y.:11y.]	[12y.:16y.]
**Antibacterials**	*Fusidic acid*	Oral suspension	44	25	30	36	9
	Josamycin	Granules for oral suspension	39	59	28	13	0
	Cefixime	Powder for oral suspension	36	8	50	39	3
	Co-amoxiclav	Powder for oral suspension	13	23	54	15	8
	Sulfamethoxazole and trimethoprim	Oral suspension	4	50	0	50	0
		Tablet	3	0	0	100	0
	Ciprofloxacin	Tablet	2	0	0	0	100
Corticosteroids	*Prednisolone*	Effervescent tablet	67	37	42	18	3
	Betamethasone	Drops for oral solution	47	43	26	28	4
	Prednisone	Tablet	4	0	25	50	25
	Hydrocortisone	Tablet	1	100	0	0	0
Antiepileptics	*Valproic acid*	Syrup	67	37	27	25	10
		Oral solution	3	67	0	33	0
	Phenobarbital	Tablet	29	48	31	21	0
	Levetiracetam	Tablet	6	83	17	0	0
	Lamotrigine	Dispersible tablet	1	0	100	0	0
Antithrombotic agents	*Acenocoumarol*	Tablet	34	6	29	53	12
Antianemic preparations	*Ferric proteinsuccinylate*	Oral solution	12	75	0	17	8
	Ferric oxide polymaltose complexes	Syrup	12	67	25	8	0
Antivirals	*Zidovudine and lamivudine*	Tablet	9	56	22	0	22
	Efavirenz	Tablet	7	29	14	14	43
	Lopinavir and ritonavir	Oral solution	5	60	20	0	20
	Lamivudine	Tablet	2	0	0	50	50

Psycholeptics	*Clobazam*	Tablet	16	25	38	38	0
	Haloperidol	Oral solution	3	33	0	67	0
	Hydroxyzine	Syrup	1	0	100	0	0
Analgesics	*Paracetamol*	Powder for oral solution	10	0	30	70	0
		Oral solution	6	17	33	50	0
		Tablet	1	0	0	100	0
	Codeine, combinations excl. Psycholeptics	Tablet	1	0	100	0	0
Ophthalmologicals	*Acetazolamide*	Tablet	14	0	7	86	7
Muscle relaxants	*Baclofen*	Tablet	13	8	23	31	38
Antimycobacterials	*Rifampicin, pyrazinamide, ethambutol and isoniazid*	Tablet	10	20	40	20	20
Mineral supplements	*Potassium chloride, combinations*	Syrup	4	50	25	0	25
	Potassium gluconate	Syrup	2	50	0	0	50
	Calcium	Syrup	2	50	0	50	0
	Calcium carbonate	Tablet	1	0	0	100	0
Constipation	*Lactulose*	Oral solution	6	33	50	17	0
	Macrogol	Powder for oral solution	2	100	0	0	0
Functional gastrointestinal disorders	*Domperidone*	Oral suspension	6	33	17	50	0
	Phloroglucinol	Tablet	1	0	0	0	100
Acid related disorders	*Omeprazole*	Effervescent tablet	3	33	33	33	0
	Alginic acid	Oral suspension	3	33	0	67	0
Urologicals	*Sildenafil*	Tablet	3	0	0	33	67
Beta blocking agents	*Propranolol*	Tablet	3	67	33	0	0
Bile and liver therapy	*Liver therapy*	Oral solution	2	0	100	0	0
Antihistamines	*Mequitazine*	Syrup	1	0	100	0	0
	Desloratadine	Oral solution	1	0	100	0	0

Antidiarrheals intestinal anti inflammatory anti infective agents	*Diosmectite*	Powder for oral suspension	2	100	0	0	0
Psychoanaleptics	*Piracetam*	Oral solution	1	100	0	0	0
Other alimentary tract and metabolism products	*Nitisinone*	Capsule	1	100	0	0	0
Diuretics	*Furosemide*	Tablet	1	0	0	100	0
Contrast media	*Diatrizoique acide*	Powder for oral solution	1	0	0	100	0
Cardiac therapy	*Digoxin*	Oral solution	1	100	0	0	0
Antiemetics and antinauseants	*Metopimazine*	Oral solution	1	0	100	0	0

© 2020 by the authors. Submitted for possible open access publication under the terms and conditions of the Creative Commons Attribution (CC BY) license (http://creativecommons.org/licenses/by/4.0/).
